# The Future of Parasitology: Challenges and Opportunities

**DOI:** 10.3389/fvets.2014.00025

**Published:** 2014-12-05

**Authors:** Hany M. Elsheikha

**Affiliations:** ^1^Neuroparasitology Laboratory, School of Veterinary Medicine and Science, Faculty of Medicine and Health Sciences, University of Nottingham, Leicestershire, UK

**Keywords:** parasitology, host–pathogen interactions, zoonoses, public health, emerging infectious disease

Parasites are being increasingly recognized as important pathogens with significant global economic, environmental, and public health impacts (1). More than three billion people worldwide are infected with one or more parasites with varying morbidity and mortality. For example, it was estimated that 740–1300 million people are infected with hookworms (*Ancylostoma duodenale, Necator americanus*), 1221–1472 million with roundworm (*Ascaris lumbricoides*), and 795–1050 million with whipworm (*Trichuris trichiura*) (2). Climate-related changes, the associated threat of vectors and vector-borne diseases (3–6), the escalating number of emerging or reemerging parasitic infections (7, 8), the alarming speed at which anti-parasitic drug resistance develops and spreads (9), and the astronomical cost of developing new anti-parasitic drugs; are just some of the challenges that make the future for treatment and control of many parasitic diseases uncertain. In the meantime, parasitology teaching and research are in a state of flux (merging, retrenchment, re-direction). This is reflected in the continued downward trend in the number of parasitology graduates and in the changing focus of the research programs, driven by limited governmental and charity funds, in industry, academic, and federal labs that used to have strong interests in solving parasitological problems. These difficulties make the scientific challenges for those trained and qualified in this discipline enormous.

Given these challenges, parasitic diseases are likely to continue to be difficult to control and, thus, new scientific knowledge will be needed to enhance control efforts. Unfortunately, knowledge gaps still exist and these need addressing in order to answer persistent questions in parasite pathobiology and control. In this article, I will discuss some of these eminent challenges and propose new concepts that could open new windows for exploration and discovery in this exciting field.

## New Technologies to Decipher Host–Parasite Interaction

A major goal for modern parasitology research is to determine signal transduction mechanisms controlling the behavior, survival, virulence, and gene expression of parasites, factors that have a crucial role in influencing the outcome of the interaction between host and parasite. The key challenge in understanding the cross-talk and communication between host and parasite is to identify characteristic aberrancies in the bio-molecular pathways and to elucidate their relationship to the progress and outcome of infection. Previous research has revealed many important aspects of parasite physiology and the complex metabolic balance between host effector molecules and pathways during parasite development and proliferation. However, previous approaches have utilized targeted analysis that provided only a snapshot and incomplete understanding of the actual dynamic of molecular events that occur rapidly during the interaction between host and parasite. This challenge can be tackled through the application of unconventional technologies and novel approaches, such as “Omics technologies,” most of which are already in progress and can be applied to study this complex process of host–parasite interaction.

## Zoonoses and Emerging Parasites

Parasitic diseases represent a significant global public health concern because many parasitic infections are zoonotic, i.e., transmitted between animals and humans (1, 2, 4, 5). Parasite zoonoses can cause a variety of symptoms in humans, from skin irritation caused by flea bites, to death from multi-organ failure as observed in advanced Lyme disease. Parasite zoonoses can result from ingesting food containing the parasite, such as meat (taeniasis, toxoplasmosis, trichinellosis); fish (anisakiosis, clonorchiosis, diphyllobothriosis), or invertebrate crustaceans (paragonimiosis); or by ingestion of the infective stage of the worm with contaminated soil (toxocariosis; echinococcosis), water, or vegetables (fascioliosis; echinococcosis; toxocariosis). Also, infection can occur via skin contact with contaminated soil/water containing infective larvae and subsequent skin penetration [e.g., cercarial dermatitis and cutaneous larva migrans in humans; *Strongyloides stercoralis* in dogs and primates, and *Halicephalobus gingivalis*, a free-living nematode that opportunistically infects horses (10) and humans (11)] or through insect vectors/intermediate hosts by ingestion (dipylidiosis) or injection by a mosquito (dirofilariosis). Some arthropods of animals such as ticks frequently attack humans and can cause tick paralysis and transmit many viral, bacterial, and protozoan diseases of animals to humans. Human dermatitis and allergic rashes caused by mites of animal origin (e.g., *Cheyletiella* spp; *Dermanyssus* spp; and *Ornithonyssus* spp) have often been reported. The potential of some zoonotic parasites to be used as biowarfare agents (e.g., certain waterborne parasites) has further alerted both the public and officials. Despite the potential for some parasites to be used as biodefense organisms, this area of research has still not been identified as a top priority.

## Exotics and Wildlife Parasitology

The fact that >70% of all emerging infections originate from wildlife reservoirs (8, 12) has driven the promotion of the “One Health” concept, an idea centered on the notion that the health of human, animal, and ecosystem are interconnected and dynamically interactive. The complexity, influence, and relevance of the human/animal/wildlife/arthropod/environment interaction on disease transmission dynamics has only begun to be explored in the last few years (13–15). The management of parasitic infections in this complex environment, coupled with the projected effects of climate change and an increasingly globalized society in which parasites do not respect geographical borders or host–species barriers, will require an increase in the allocation of research and development funding and a multidisciplinary approach. Parasitologists are uniquely suited to address this research area especially with the availability of an array of new surveillance tools, expanded bioinformatic/mathematical modeling, and global positioning systems.

To tackle these current and future challenges, we need to advance scientific knowledge through the application of novel approaches. Likewise, for an effective translation of scientific discoveries and applications in the field of parasitology, we need an outlet to enhance effective communication among scientists with different backgrounds and who share a common interest in parasitology. This was the drive for launching a Parasitology specialty section in the journal *Frontiers in Veterinary Science*, an international, open-access, peer-reviewed, online journal. It gives me great pleasure to announce the inauguration of this new Parasitology specialty section with the ethos of publishing the most impactful and interesting research discoveries in basic and fundamental areas of parasitology. The Parasitology specialty section will publish original research articles, reviews of latest developments in specific subject areas and forward-looking perspectives.

Specific areas of interest include, but are not limited to, mechanisms of parasite pathogenesis, experimental models of infection, host resistance/susceptibility, parasite immunity, cellular responses to parasitic infection, parasite genomics, genetic diversity, population genetics, and evolution. Also, the section will consider manuscripts presenting innovative and improved methods that have the potential to advance parasitology research with broad interests. Especially welcome are cutting-edge clinical parasitology research studies that employ state-of-the-art methods, are driven by fundamental insights and interdisciplinary approaches, and aim to improve our understanding of the underpinning mechanisms and therapeutic applications of anti-parasitic chemotherapy and vaccines.

As the founding Specialty Chief Editor for Parasitology *Frontiers in Veterinary Science*, I have accepted the challenges and responsibilities of developing a premier professional journal in Parasitology. The editorial team will strive to maintain excellence while improving the efficiency of the review process and taking advantage of the latest online technology. All editorial board members are leaders in their respective scientific areas and are willing to devote their time and expertise to build a journal of excellence to serve the Parasitology research community, and life sciences in general, with the ultimate goal of improving animal and human lives. I hope that the Parasitology section will appeal to readers with veterinary, medical, and life science interests.

To conclude, novel developments in the control of parasitic diseases can be facilitated by a better understanding of host–parasite interaction. However, one of the most pressing challenges is how to leverage the power of scientific knowledge to address the challenge of unraveling the complex interplay of host defense machinery and parasite signaling/effector molecules that regulate host–parasite interaction. More advancement will come from new technological improvements and new approaches. In addition to revealing fundamental cellular mechanisms, future studies of host–parasite relationships will enhance our understanding of parasite pathogenesis, and may lead to improved diagnostics and treatments for parasitic infections. I look forward to receiving your exciting research contributions in these areas. Your ideas and suggestions for making the journal highly impactful are welcomed and will be always valued.

## Conflict of Interest Statement

The author declares that the research was conducted in the absence of any commercial or financial relationships that could be construed as a potential conflict of interest.
